# 3D C-arm navigated acromioclavicular joint stabilization

**DOI:** 10.1007/s00402-023-05112-5

**Published:** 2023-11-08

**Authors:** Alexander Böhringer, Florian Gebhard, Christoph Dehner, Alexander Eickhoff, Raffael Cintean, Carlos Pankratz, Konrad Schütze

**Affiliations:** Department of Trauma Hand and Reconstructive Surgery, Ulm University, Albert-Einstein-Allee 23, 89081 Ulm, Germany

**Keywords:** Tossy, Rockwood, AC joint separation, Tightrope, 3D C-arm navigation, Shoulder imaging

## Abstract

**Introduction:**

Surgical treatment options for acromioclavicular joint separations are varied. Frequently, suspension devices (SD) are inserted for stabilization under arthroscopic view. This study investigates the feasibility and accuracy of three-dimensional (3D) digital-volume-tomography (DVT) C-arm navigated implantation with regard to the general trend toward increasingly minimally invasive procedures.

**Materials and methods:**

The implantation of a TightRope^®^ suture button system (SD) via a navigated vertical drill channel through the clavicle and coracoid was investigated in 10 synthetic shoulder models with a mobile isocentric C-arm image intensifier setup in the usual parasagittal position. Thereby, in addition the placement of an additive horizontal suture cerclage via a navigated drill channel through the acromion was assessed.

**Results:**

All vertical drill channels in the Coracoclavicular (CC) direction could be placed in a line centrally through the clavicle and the coracoid base. The horizontal drill channels in the Acromioclavicular (AC) direction ran strictly in the acromion, without affecting the AC joint or lateral clavicle. All SD could be well inserted and anchored. After tensioning and knotting of the system, the application of the horizontal AC cerclage was easily possible. The image quality was good and all relevant structures could be assessed well.

**Conclusion:**

Intraoperative 3D DVT imaging of the shoulder joint using a mobile isocentric C-arm in the usual parasagittal position to the patient is possible. Likewise, DVT navigated SD implantation at the AC joint in CC and AC direction on a synthetic shoulder model. By combining both methods, the application in vivo could be possible. Further clinical studies on feasibility and comparison with established methods should be performed.

## Introduction

Traumatic acromioclavicular (AC) joint separations are common shoulder injuries, representing 4–12% of all shoulder girdle injuries. They often occur in amateur sports such as skiing, cycling, or football due to direct impact, with an incidence of 3–4 cases per 100,000 inhabitants per year [1]. Probably the most common classification of shoulder joint separations is the Rockwood classification, developed in 1984 from the Tossy classification published in 1963 and currently modified by a consensus procedure of the European Shoulder Associates [2, 3]. Type 1 injuries have sprained but still intact acromioclavicular (AC) and coracoclavicular (CC) ligaments. Type 2 injuries have torn AC ligaments and sprained or at most partially torn CC ligaments. Type 3a injuries have completely torn AC and CC ligaments with significant vertical elevation of the lateral clavicle. Type 3b injuries have horizontal instability in addition to vertical instability. In type 4 injuries, the lateral clavicle is firmly displaced dorsally. In type 5 injuries, the lateral clavicle is displaced cranially by 100–300%. And in the very rare type 6 injuries the lateral clavicle is hooked inferiorly [4]. There is general consensus for conservative treatment of type 1 and 2 injuries and surgical treatment of type 4–6 injuries. For type 3 injuries, there is a relative indication for surgery [5], 3a more conservative and 3b more surgical. The dividing line between acute and chronic injuries is set at 3 weeks. More than 150 different surgical therapy options are known [5]. Equivalent and most frequently used are the conventional hook plate and the already well-established suspension devise (SD) procedure [6, 7]. With this method, implant removal is no longer necessary and with regard to the trend toward increasingly minimally invasive surgical methods, the SD can be placed under arthroscopic guidance since the year 2001 [8]. Thus, for acute injuries, arthroscopically assisted anatomic reconstruction with a SD is favored and no biological augmentation is recommended, whereas for chronic injuries, biological reconstruction of the CC and AC ligaments with a tendon graft can be performed [2]. In addition to SD stabilization of the CC ligaments, the application of a horizontal AC cerclage is recommended by some authors [9–12]. For better intraoperative visual assessment of the shoulder joint, digital volume tomography (DVT) can be performed using a mobile C-arm [13–15]. Under laboratory conditions, Stübig et al. were able to demonstrate more accurate drill channel placement CC on nine cadaver shoulders using navigation with a non-isocentric flat detector C-arm in three dimensions (3D) than under conventional two-dimensional (2D) radiographic control [16]. With this study on 10 shoulder models, we investigate the feasibility and accuracy of 3D isocentric C-arm navigated SD implantation CC and AC under everyday conditions in the operating room.

## Material and methods

**Fig. 1:**
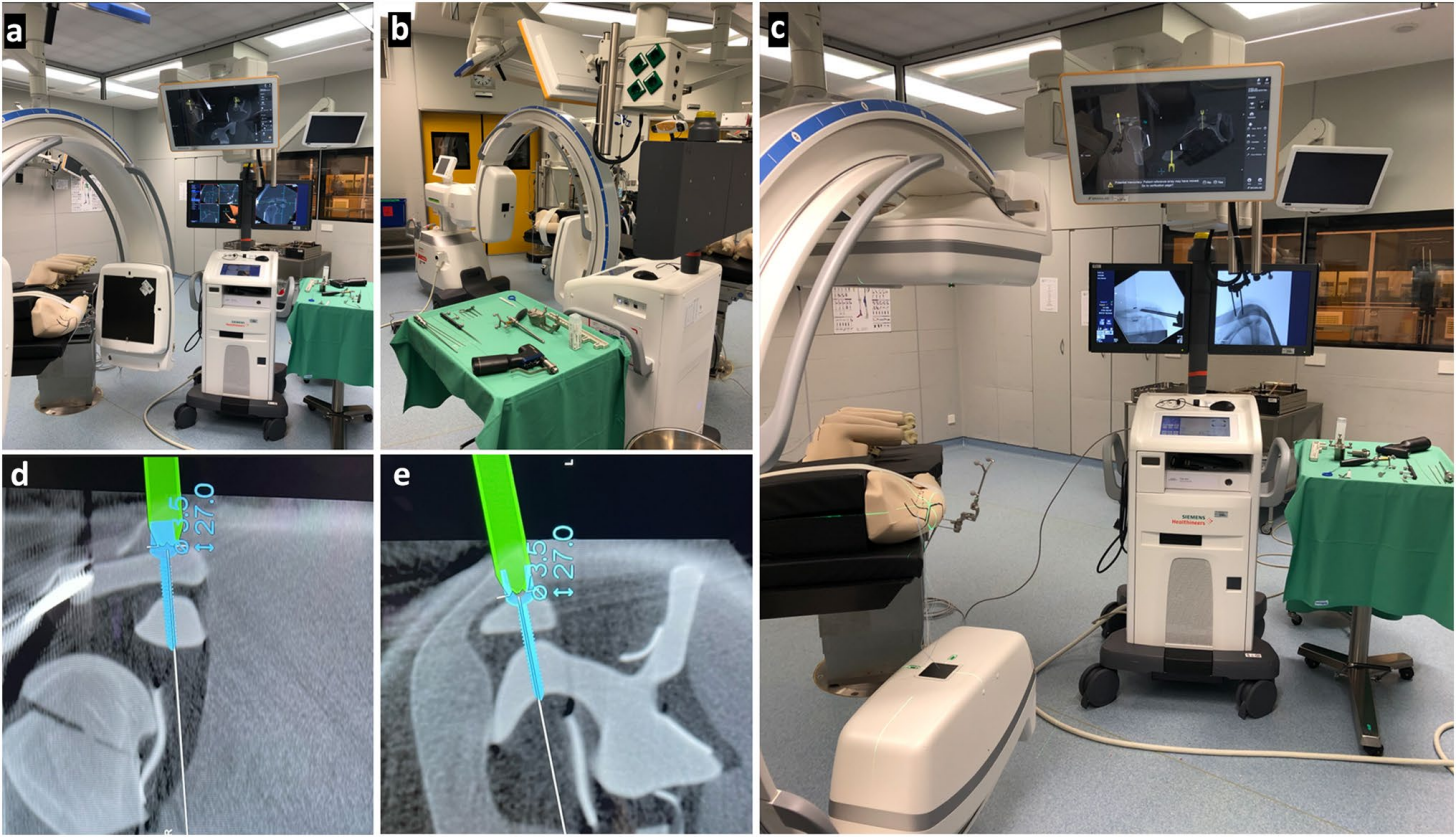
System setup in the operating room. **a**–**c** a synthetic shoulder model is attached to the operating table. The mobile C-arm is placed in parasagittal position at the head end. The navigation camera is pointed at the references on the model and the C-arm. The navigation monitor still shows images of the first DVT scan with a planned CC drill channel. On the X-ray monitor, the already implanted SD is visible in two planes. The drill, wires, and pointers are on the instrument table. **d**–**e** the planning of the navigated drill channel in CC direction is shown enlarged in two planes (coronal and sagittal)

**Fig. 2:**
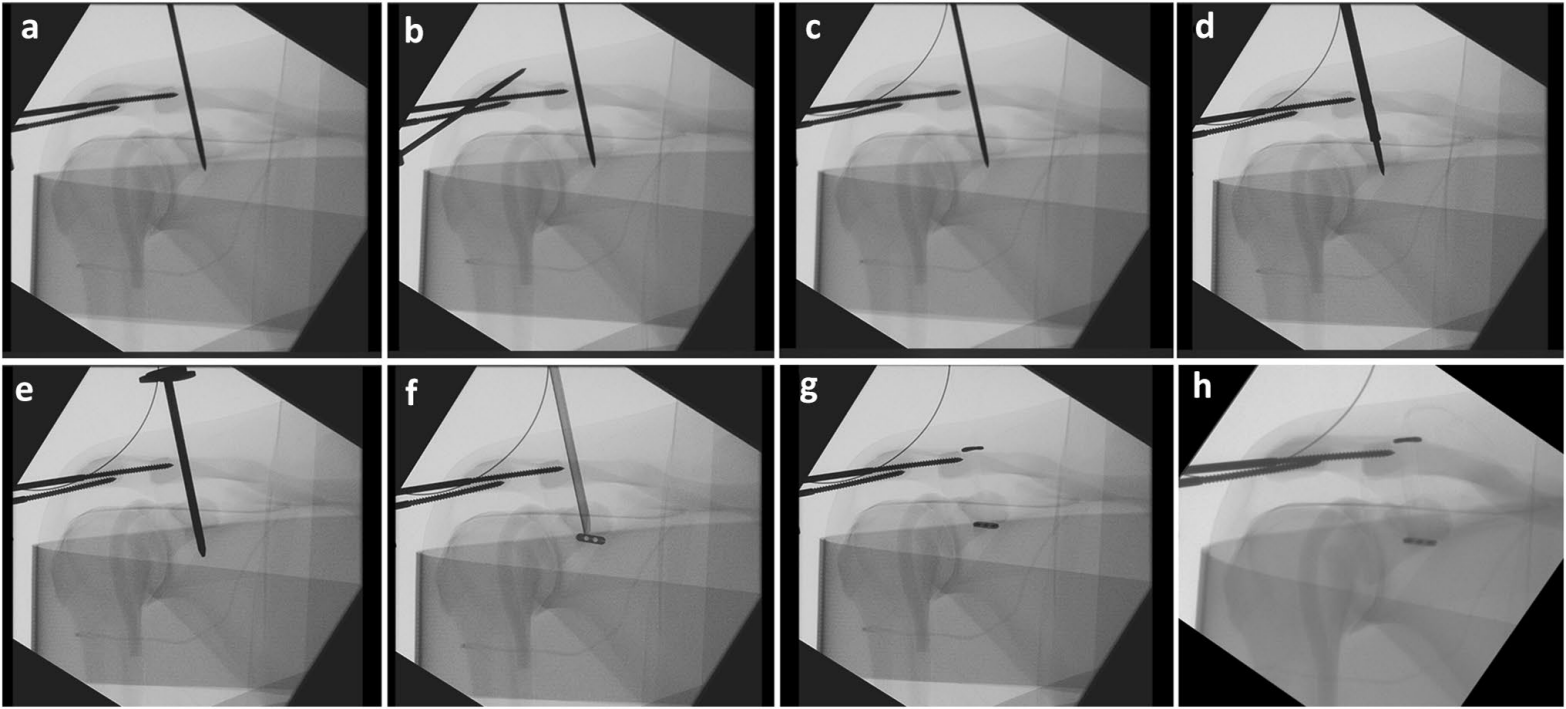
Navigated drilling and implantation of the SD. **a** the reference is attached to the lateral acromion margin, temporarily fixing the AC joint and the CC drill channel is already being prepared with a lying drill wire. **b** the AC drill channel is created. **c** the drill wire in the acromion is replaced by a wire loop through which the suture loop is pulled later. **d** the lying CC drill wire is overdrilled using a cannulated drill. **e** a trocar is introduced CC. **f** the SD is inserted through the trocar. **g** the SD is anchored below the coracoid and above the clavicle. **h** the SD is knotted and then a thread end will be pulled laterally through the acromion using the wire loop

For this study, 10 intact right shoulder models from SYNBONE® were used (part number PR0720.1—Right shoulder with vise attachment, scapula, clavicle, humerus, ligaments, biceps tendon, axillary nerve, and soft tissue). The models are manufactured with a specially formulated polyurethane foam comprising of a cancellous inner core and a harder outer shell simulating the cortical bone. They are primarily developed for orthopedic surgical education and are designed to provide the feeling of working with humanlike bone. Similar forces are required to saw, tap, plate, and drill these models. The shoulder models were fixed in beach chair position on a carbon table under everyday conditions in the operating room. Subsequently, closed retention of the AC joint with a Kirschner wire was performed percutaneously under 2D radiographic control using a mobile isocentric C-arm image intensifier (Siemens^®^ Cios Spin) placed in the usual parasagittal position. A second wire was inserted parallel to the first to attach the navigation reference to the lateral acromion. Then the first DVT scan was performed and the navigation system was integrated (Brainlab “Buzz,” consisting of hardware (instruments, references, cameras, and screens) and software (Backbone 1.6.2.54, Backbone Viewer 1.6.2.578, Brainlab Buzz 1.0.0.12, and Brainlab Nodemaster 1.6.0.48)). First, the AC joint position was checked. Then the CC drill channel was precisely planned on the navigation monitor. Subsequently, the drill channel was created in a navigated fashion through a stab incision above the clavicle on the shoulder model. The suspension device (SD, Arthrex TightRope^®^ suture button system) was then guided through the drill channel and anchored below the coracoid base and tightened above the clavicle. In addition, the AC drill channel was planned and also created using navigation. One suture end of the fixed CC SD was then passed laterally through the AC drill canal with a nitinol wire^®^ (Arthrex) and returned close to the bone above the acromion under the soft tissues to be reknotted with the other suture end under tension. Finally, another DVT scan was made for visual control and assessment of the drill channels and the inserted SD. In Figs. 1 and 2 the system setup, the drill channels planning on the monitor and the performance of the procedure under imaging control are shown step by step.

**Fig. 3:**
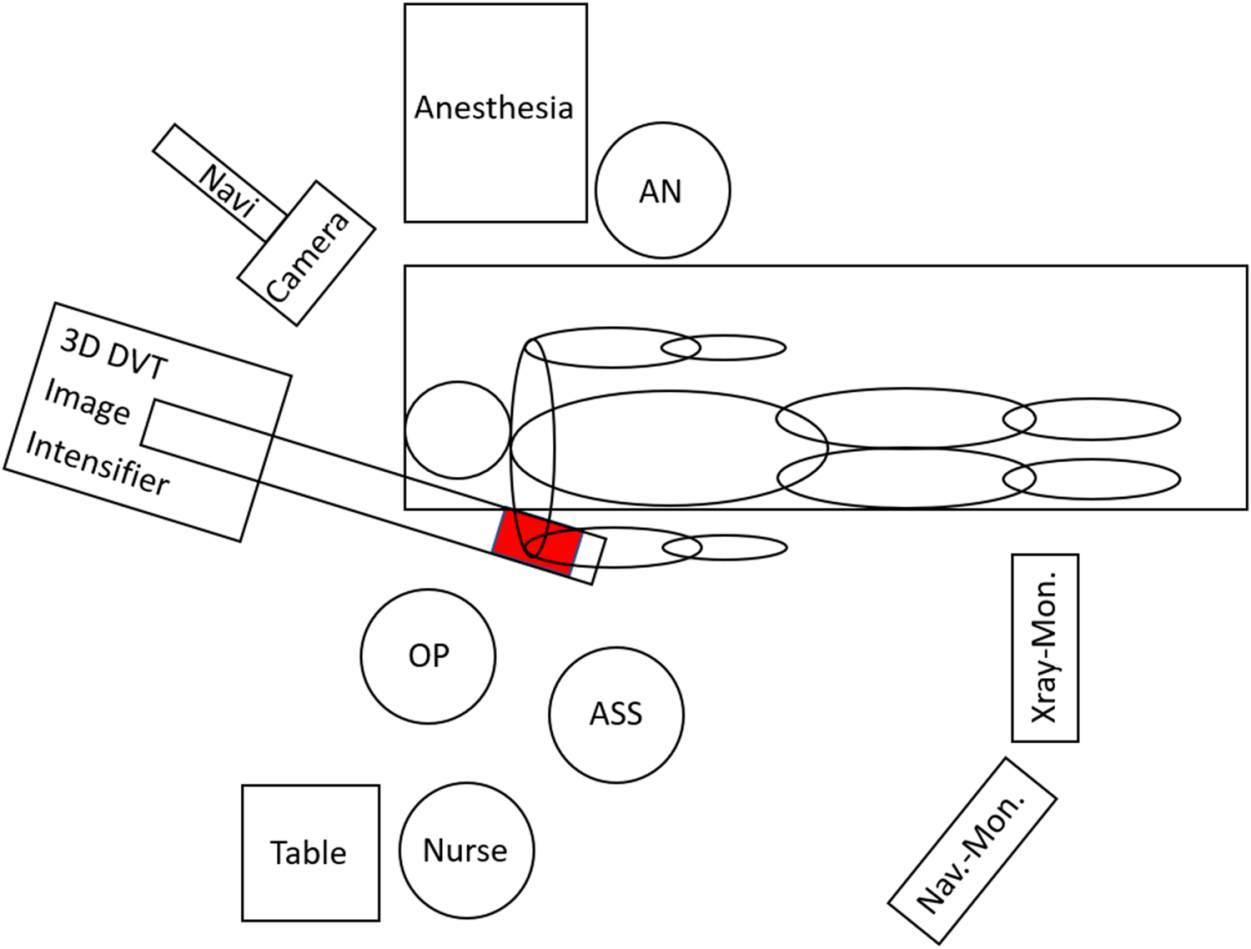
Illustrated in a sketch. Shown is the arrangement of the mobile C-arm and navigation camera to the shoulder models on the operating table in the center. Surgeon (OP), assistant (ASS), nurse, anesthesia (AN), and monitors around. The area marked in red represents the imaged body region (right shoulder with complete AC joint) between the X-ray tube and detector of the C-arm

The system setup in the operating room was identical to that of the study “intraoperative 3D imaging in plate osteosynthesis of proximal humerus fractures” [15]. The same operating table and the same C-arm were used in exactly the same position to each other as shown in Fig. 3.

**Fig. 4:**
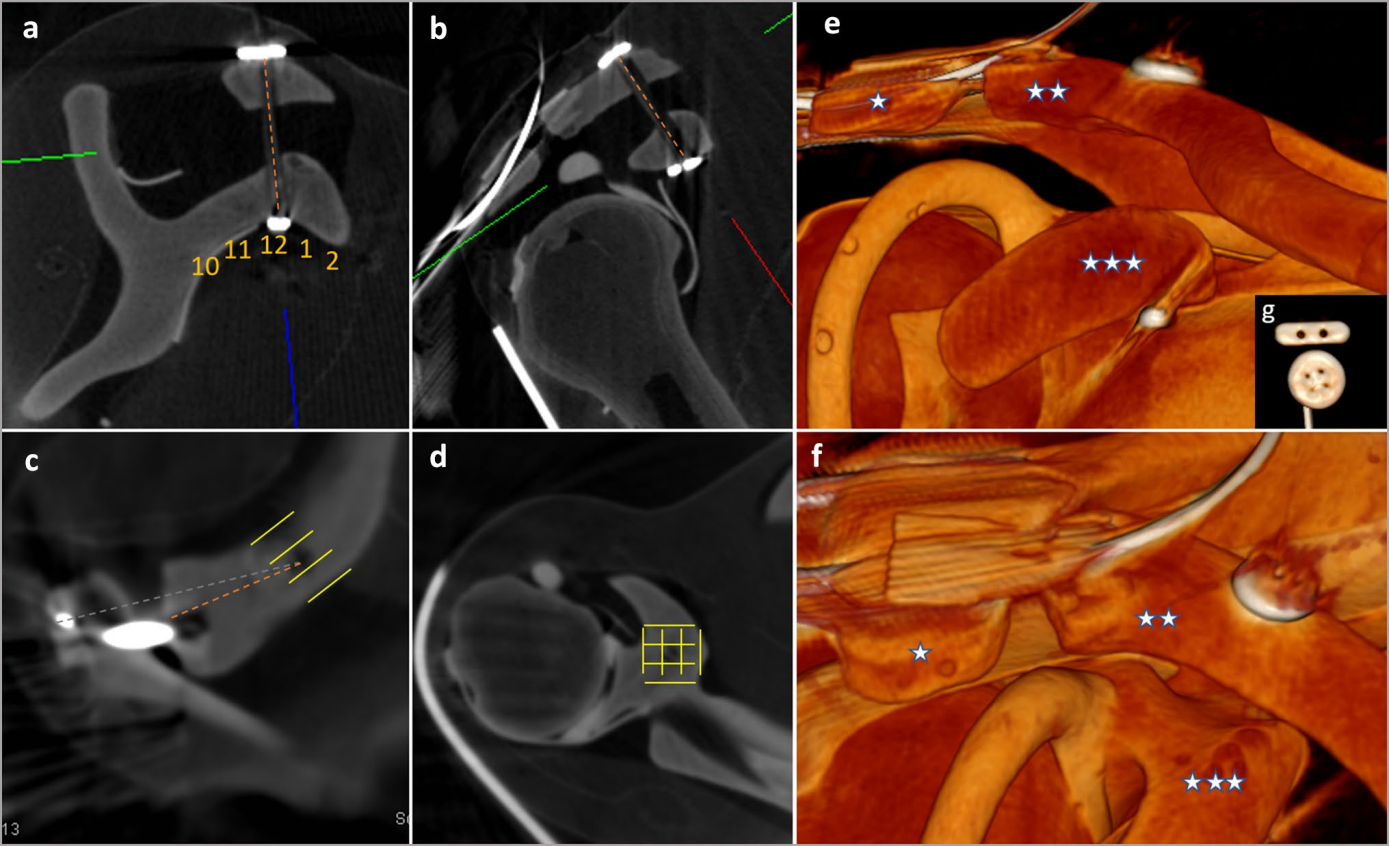
Intraoperative DVT images of the examined models for control and measuring of the inserted SD in multiplanar view (**a**–**d**) and 3D reconstruction (**e**–**g**). **a** Sagittal plane to measure the position and length (dashed line) of the CC drill channel and assess the button position above the clavicle and below the coracoid (here the drill channel is centered in the clavicle and the lower button is directly at the tip of the coracoid arch at 12 o’clock) directly on the bone. **b** Coronal plane for measuring and evaluating the CC and AC drill channel as well as the button position (the central position of the CC drill channel and the button placed directly underneath the coracoid as well as the correctly positioned wire loop in the AC drill channel without affecting the lateral clavicle are confirmed here). **c** Axial plane to evaluate the central position of the drill channel entrance at the surface of the clavicle (yellow parallel lines) as well as the distance from here to the lateral end of the clavicle (orange dashed line) as well as the direction of the AC drill channel (gray dashed line from the wire loop to the center of the drill hole). **d** Axial plane at the level of the coracoid surface to determine the central drill channel position in anterior–posterior and medial–lateral direction (yellow grid lines). **e** and **f** 3D reconstruction in the direction of view from medioventral and cranioventral for three-dimensional assessment of the AC joint position (acromion = one star, lateral clavicle = two stars and coracoid = three stars) and the implant position (inserted wire loop in the AC drill channel, attached button above the clavicle and below the coracoid). **g** Hard paned 3D image reconstruction with direct view to the two CC titanium buttons from cranial to caudal

Postoperatively, the intraoperative DVT images were evaluated with the DeepUnity^®^ program (Diagnost 1.2.0.1, Dedalus) as shown in Fig. 4. To determine the accuracy of the navigated drill holes, the drill channels were measured in length and position in the clavicle, coracoid, and acromion. In addition, the implant position was assessed. Furthermore image quality was evaluated using a modified VAS (visual analog scale) and points system by two independent investigators (both senior orthopedic trauma residents) [17, 18]. The general image quality, the delineation of corticalis, cancellous bone and joint surface, as well as the occurrence of disturbing metal artifacts and the general accessibility of clinically relevant structures and implants were examined. The results are shown in Tables 1, 2, 3, 4.

**Table 1:** Results of the visual analog scale for assessing intraoperative 3D DVT image quality

VAS (0–10 = 0 for poor and 10 for excellent image quality)						
Synbone^®^ model	Image quality	Cortical bone	Cancellous bone	Articular surface	No artifacts	Clinical assessment
1	9	10	9.5	8	8	10
2	8.5	9.5	9	8	7.5	10
3	9	9	9	8	7.5	9.5
4	8	9	8.5	7.5	7	9
5	8	9.5	9	8	8	9.5
6	8	9	9	7	7	9
7	8.5	9.5	9	8	8	9.5
8	9	10	9.5	8.5	8	10
9	8.5	9	9	7.5	7.5	9.5
10	9	9.5	9.5	8	8	10
On average	8.6	9.4	9.1	7.9	7.7	9.6

In general, the subjectively assessed image quality and clinical accessibility of the relevant structures are presented. In addition, the quality is evaluated based on the possible delineation of corticalis, cancellous bone, articular surface, and the presence of interfering implant artifacts

**Table 2:** Explanation and results of the points system for assessing intraoperative 3D DVT image quality

Points system (1–5 = 1 for poor and 5 for excellent image quality)						
Synbone^®^ model	Subjective image quality total	Delineation of cortical bone	Delineation of cancellous bone	Delineation of articular surfaces	No artifacts	Clinical assessment total
1	5	5	5	5	4.5	5
2	5	4.5	4.5	5	4.5	5
3	5	4.5	4.5	5	4	5
4	4.5	4	4	4	4	5
5	5	4.5	4.5	5	4	5
6	4.5	4	4	4	4	5
7	5	4.5	4	5	4	5
8	5	5	5	5	4.5	5
9	5	4	4	4.5	4	5
10	5	5	5	5	4.5	5
On average	4.9	4.5	4.5	4.8	4.2	5

In general, the subjectively assessed image quality and clinical accessibility of the relevant structures are presented. In addition, the quality is evaluated based on the possible delineation of corticalis, cancellous bone, articular surface, and the presence of interfering implant artifacts

**Table 3:** Results of the drill channel measurements on the DVT images of the ten synthetic shoulder models

Drill holes (DHs)							
Synbone^®^ shoulder model number	Distance clavicle DH to AC joint in mm	Position clavicle DH anterior/center/posterior	Position coracoid DH anterior/center/posterior	Position coracoid DH medial/center/lateral	Distance CC in mm	AC drill channel in line with clavicle DH y/n	AC drill channel affecting AC joint y/n
1	25	Center	Center	Center	29	y	n
2	23	Center	Center	Center	30	y	n
3	25	Center	Center	Center	29	y	n
4	28	Center	Center	Center	30	y	n
5	26	Center	Center	Center	30	y	n
6	25	Center	Center	Center	31	y	n
7	26	Center	Center	Center	30	y	n
8	27	Center	Center	Center	28	y	n
9	26	Center	Center	Center	27	y	n
10	28	Center	Center	Center	30	y	n

**Table 4:** Assessment of the exact implant position and visibility on the DVT images

Suspension device (SD)						
Synbone^®^ shoulder model number	SD CC feasible y/n	Cerclage AC feasible y/n	Button flush y/n	Button position below the coracoid between 10 and 2 o’clock	Visible number of holes in the clavicle button	Visible number of holes in the coracoid button
1	y	Y	y	12	4	2
2	y	y	y	12	4	2
3	y	y	y	12	4	2
4	y	y	y	12	4	2
5	y	y	y	12	4	2
6	y	y	y	12	4	2
7	y	y	y	12	4	2
8	y	y	y	12	4	2
9	y	y	y	12	4	2
10	y	y	Y	12	4	2

## Results

First, it can be noted that the image quality was good and all relevant structures (clavicle, acromion, coracoid, AC joint position, titanium button, and wire), see Tables 1 and 2, could be assessed perfectly. Even the correct number of holes in the titanium buttons could be detected without any issues (see Fig. 4, in implant artifact assessment, the discriminatory power allows reliable assessment of the 1-mm-diameter holes in the titanium buttons).

The measurement results of the navigated SD implantations are shown in Tables 3 and 4. All ten vertical drill channels in the CC direction could be placed in a line centrally through the clavicle and the coracoid base. The mean distance from the drill hole entrance in the clavicle to the AC joint was 26 mm. The CC drill channel length was 29 mm on average. The horizontal drill channels in the AC direction ran strictly in the acromion, without affecting the AC joint or lateral clavicle. Moreover, they were directed in a line toward the respective vertical CC drill hole entrance at the clavicle. All SD could be easily inserted into the corresponding CC drill channel and anchored below the coracoid. After tensioning and knotting of the system, the application of the horizontal cerclage was always well possible. After implantation, the titanium buttons all appeared to be in direct contact with the bone. The lower button could be placed almost parallel to the upper one at the apex of the coracoid arch at 12 o’clock.

## Discussion

Some studies in recent years report persistent complication risks even with the two most established procedures for surgical treatment of severe AC joint injuries, the hook plate and the SD. Secondary loss of reduction, persistent instability, implant dislocation, bone erosion, pain, and postoperative joint stiffness are most common [5–7, 19–28]. Thus, further research is needed. In general, as here, there is a general trend toward increasingly minimally invasive and accurate surgical methods with reconstruction as anatomic as possible [1, 2].

The aim of this study was to demonstrate the feasibility of DVT navigated SD implantation at the AC joint in CC and AC direction under everyday surgical conditions using a synthetic shoulder model. Arthroscopically assisted SD implantations are already well established and are currently widely used as state of the art [1, 2]. DVT navigated SD implantation would be even more minimally invasive, but by now this has only been demonstrated in two studies on human cadaver shoulders [16, 29].

In one study with human cadaver shoulders and another study with patients, Theopold et al. showed that arthroscopically assisted CC drill channel placement is more precise than open drilling with a targeting device [30, 31]. However, Stübig et al. were able to show in their two studies that DVT navigated CC drill channel placement is even more precise than arthroscopically assisted or under 2D radiographic control [16, 29].

Some authors recommend AC cerclage in addition to CC SD implantation to stabilize the horizontal and axial AC joint injury components [9–12, 32–37]. In the two studies by Stübig et al., only DVT navigated CC SD stabilization was demonstrated. The possibility of placing an AC cerclage was not investigated [16, 29]. The method of DVT navigated SD implantation described by Stübig et al. has neither been studied in vivo nor has the procedure been introduced into clinical practice so far.

In our present DVT navigated SD study, the conditions were adapted to everyday surgical practice and the system setup in the operating room was identical to the in vivo study on intraoperative 3D C-arm visualization of the shoulder joint [15]. As in the two studies by Stübig et al., the CC drill channels in our study could be placed very precisely in a line always centered on the clavicle and the coracoid [16]. Furthermore, in our study, an additional drill channel could be accurately created through the acromion for horizontal stabilization of the AC joint. The insertion of SD implants through the navigated CC drill channels was briefly mentioned in the study by Stübig et al. but not further described or visually demonstrated [16]. However, the reliably feasible insertion and anchorage of these implants could be shown in detail in our study. In addition, we were able to demonstrate the simple application of AC suture cerclage through a navigated acromion drill channel.

In a recently published study, we have already demonstrated, contrary to previous opinion, that it is possible to perform intraoperative 3D DVT on the shoulder with an isocentric mobile C-arm in the usual parasagittal position to the patient [15, 38]. In a retrospective evaluation of these images, we were able to additionally demonstrate the imageability of the entire AC joint (lateral clavicle, coracoid, and acromion) with regard to the current question. The image quality in our present study on synthetic shoulder models as well as in the re-evaluated retrospective shoulder DVT images of our previous study was better than partly described in the literature [39]. This could be related to the use and arrangement of a mobile isocentric C-arm, which is used to image only the shoulder region and does not require irradiation of the entire patients thorax. For the time of the DVT recording, the entire surgical team including the anesthesia can temporarily leave the operating room and thus avoid their own radiation exposure.

The feasibility of intraoperative 3D DVT imaging of the shoulder in vivo has only been published so far for proximal humerus fractures and for accurate positioning of the baseplate in reverse shoulder arthroplasty [13–15, 39]. However, in combination with navigation it might also be useful for accurate assessment of AC joint repositioning, accurate drill channel placement, and control of SD implantation.

Regarding limitations, this is a feasibility study on a synthetic shoulder model. The number of ten shoulder models is small. No clinical parameters on complaints or function were recorded. No evaluation of comparisons between different methods, such as arthroscopically assisted SD implantation or the hook plate, was made and no biomechanical studies were performed.

## Conclusion

Intraoperative 3D DVT imaging of the shoulder joint using a mobile isocentric C-arm in the usual parasagittal position to the patient is possible. Likewise, DVT navigated SD implantation at the AC joint in CC and AC direction on the shoulder model. By combining both methods, the application in vivo could be possible. Thus, 3D-imaging could possibly improve the precision of drill holes or make the arthroscopic control in this operation obsolete.

Next, a clinical study on the feasibility of navigated SD implantation in vivo should be performed. This could be followed by further clinical studies comparing the procedure with the two established methods of arthroscopically assisted SD implantation and the clavicle hook plate.

## Data Availability

All the authors decided that the data and material will not be deposited in a public repository.
